# Supplementary material for: Quantifying conformational changes in the TCR:pMHC-I binding interface
Source: Front Immunol. 2024 Dec 2;15:1491656. doi: 10.3389/fimmu.2024.1491656 (PMC11646856; doi:10.3389/fimmu.2024.1491656)
Supplement: Supplementary file 1 [file DataSheet1.pdf]

Supplementary Material for ‘Quantifying conformational changes in the TCR:pMHC-I binding interface’

1 CORRELATION BETWEEN CONFORMATIONAL CHANGE AND AFFINITY

We were interested in whether there was a correlation between the amount of conformational change in the binding interface of T cell antigen receptor (TCR):class I peptide-MHCs (pMHC-Is) between *apo* and *holo* states and the affinity of the interaction. We combined the results of our analysis of crystal structures with affinity measures available in the ATLAS dataset Borrman et al. (2017). Shown in Fig. S9 and Fig. S10, we plotted the changes in conformation, measured as root mean squared deviation (RMSD), versus the affinity values, measured as the equilibrium dissociation (K_d), and measured the correlation. Only the CDR2α, CDR3α, and CDR3β loops showed a small correlation to affinity ($R^2 > 0.00$). The other loops show very little to no correlation. On the pMHC-I side, neither the movement in peptide, major histocompatability complex (MHC) TCR contacting regions, nor the rest of the MHC antigen binding domain showed any correlation to affinity. This analysis suggests that although there are slight correlations between conformational change and affinity, the trends are subtle and one is not indicative of the other.

2 CORRELATION OF CDR LOOP AND PEPTIDE LENGTHS TO CONFORMATIONAL CHANGE

Probing the underlying causes of conformational change in the complementarity determining region (CDR) loops and peptides, we investigated the role length plays in allowing for conformational change. Shown in Fig. S11A, bulk CDR movements are weakly correlated to RMSD changes between *apo* and *holo* states ($R^2 = 0.11$). However, Fig. S11B shows a stronger correlation to the deformation effect of loops ($R^2 = 0.35$). The increased correlation makes sense since other parts of the protein can drive the conformational changes from the framework regions, but when the loops are aligned together, the only differences can be driven by changes in the loops themselves, implying the loop length has more of an effect. Looking at the correlation between peptide length and conformational change in Fig. S11C, we see that there is a moderate correlation ($R^2 = 0.17$). From these results, it seems both loop and peptide lengths have some impact on the inherent flexibility of these entities, but other factors also likely modulate the flexibility such as amino acid composition and interactions with other domains Milighetti et al. (2024).

3 SUPPLEMENTARY TABLES AND FIGURES

Table S1: Breakdown of the data used in the analysis of *apo* and *holo* forms of TCR and pMHC structures. In total, 391 structures are coming from 301 PDB entries.

PDB ID	Structure Type	α-chain ID	β-chain ID	Antigen Chain ID	MHC Chain ID
1b0g	pMHC-I	-	-	C	A
1b0g	pMHC-I	-	-	F	D
1bii	pMHC-I	-	-	P	A

Continued on next page

Table S1: Breakdown of the data used in the analysis of *apo* and *holo* forms of TCR and pMHC structures.

PDB ID	Structure Type	α-chain ID	β-chain ID	Antigen Chain ID	MHC Chain ID
1ddh	pMHC-I	-	-	P	A
1duz	pMHC-I	-	-	C	A
1duz	pMHC-I	-	-	F	D
1e28	pMHC-I	-	-	C	A
1ffn	pMHC-I	-	-	C	A
1ffn	pMHC-I	-	-	F	D
1fzj	pMHC-I	-	-	P	A
1fzm	pMHC-I	-	-	P	A
1hhi	pMHC-I	-	-	C	A
1hhi	pMHC-I	-	-	F	D
1hhk	pMHC-I	-	-	C	A
1hhk	pMHC-I	-	-	F	D
1hoc	pMHC-I	-	-	C	A
1i4f	pMHC-I	-	-	C	A
1jf1	pMHC-I	-	-	C	A
1jht	pMHC-I	-	-	C	A
1kj3	pMHC-I	-	-	P	H
1kj3	pMHC-I	-	-	Q	I
1kpu	pMHC-I	-	-	P	A
1leg	pMHC-I	-	-	P	A
1lek	pMHC-I	-	-	P	A
1m05	pMHC-I	-	-	E	A
1m05	pMHC-I	-	-	F	C
1n5a	pMHC-I	-	-	C	A
1n5a	pMHC-I	-	-	F	D
1n5a	pMHC-I	-	-	I	G
1n5a	pMHC-I	-	-	L	J
1nan	pMHC-I	-	-	M	H
1s7u	pMHC-I	-	-	C	A
1s7u	pMHC-I	-	-	F	D
1s7u	pMHC-I	-	-	I	G
1s7u	pMHC-I	-	-	L	J
1s9w	pMHC-I	-	-	C	A
1t1w	pMHC-I	-	-	C	A
1t20	pMHC-I	-	-	C	A
1t21	pMHC-I	-	-	C	A
1t22	pMHC-I	-	-	C	A
1wby	pMHC-I	-	-	C	A
1yn6	pMHC-I	-	-	C	A
1zhk	pMHC-I	-	-	C	A
1zhl	pMHC-I	-	-	C	A
2av1	pMHC-I	-	-	C	A
2av1	pMHC-I	-	-	F	D
2av7	pMHC-I	-	-	C	A
2av7	pMHC-I	-	-	F	D
2bss	pMHC-I	-	-	C	A
2c7u	pMHC-I	-	-	C	A
2c7u	pMHC-I	-	-	F	D

Continued on next page

Table S1: Breakdown of the data used in the analysis of *apo* and *holo* forms of TCR and pMHC structures.

PDB ID	Structure Type	α-chain ID	β-chain ID	Antigen Chain ID	MHC Chain ID
2clv	pMHC-I	-	-	C	A
2clv	pMHC-I	-	-	M	H
2clz	pMHC-I	-	-	C	A
2clz	pMHC-I	-	-	M	H
2f74	pMHC-I	-	-	C	A
2f74	pMHC-I	-	-	F	D
2gt9	pMHC-I	-	-	C	A
2gt9	pMHC-I	-	-	F	D
2gtz	pMHC-I	-	-	C	A
2gtz	pMHC-I	-	-	F	D
2guo	pMHC-I	-	-	C	A
2guo	pMHC-I	-	-	F	D
2mha	pMHC-I	-	-	E	A
2mha	pMHC-I	-	-	F	C
2v2w	pMHC-I	-	-	C	A
2v2w	pMHC-I	-	-	F	D
2vaa	pMHC-I	-	-	P	A
2vll	pMHC-I	-	-	C	A
2vll	pMHC-I	-	-	F	D
2x4r	pMHC-I	-	-	C	A
2x4r	pMHC-I	-	-	F	D
3dx8	pMHC-I	-	-	C	A
3ecb	pMHC-I	-	-	P	A
3gso	pMHC-I	-	-	P	A
3h7b	pMHC-I	-	-	C	A
3h7b	pMHC-I	-	-	F	D
3h9h	pMHC-I	-	-	C	A
3h9h	pMHC-I	-	-	F	D
3hpj	pMHC-I	-	-	C	A
3hpj	pMHC-I	-	-	F	D
3ixa	pMHC-I	-	-	C	A
3ixa	pMHC-I	-	-	F	D
3kpp	pMHC-I	-	-	C	A
3kpq	pMHC-I	-	-	C	A
3kww	pMHC-I	-	-	C	A
3mre	pMHC-I	-	-	P	A
3mrm	pMHC-I	-	-	P	A
3nfn	pMHC-I	-	-	C	A
3pwl	pMHC-I	-	-	C	A
3pwl	pMHC-I	-	-	F	D
3qfd	pMHC-I	-	-	C	A
3qfd	pMHC-I	-	-	F	D
3quk	pMHC-I	-	-	C	A
3quk	pMHC-I	-	-	F	D
3sko	pMHC-I	-	-	C	A
3tbs	pMHC-I	-	-	C	A
3tbs	pMHC-I	-	-	F	D
3tby	pMHC-I	-	-	C	A

Continued on next page

Table S1: Breakdown of the data used in the analysis of *apo* and *holo* forms of TCR and pMHC structures.

PDB ID	Structure Type	α-chain ID	β-chain ID	Antigen Chain ID	MHC Chain ID
3tby	pMHC-I	-	-	F	D
3tby	pMHC-I	-	-	I	G
3tby	pMHC-I	-	-	L	J
3utq	pMHC-I	-	-	C	A
3vfm	pMHC-I	-	-	C	A
3vfn	pMHC-I	-	-	C	A
3vfo	pMHC-I	-	-	C	A
3vfp	pMHC-I	-	-	C	A
3vxn	pMHC-I	-	-	C	A
3vxo	pMHC-I	-	-	C	A
3vxo	pMHC-I	-	-	F	D
3vxp	pMHC-I	-	-	C	A
3vxp	pMHC-I	-	-	F	D
3x13	pMHC-I	-	-	C	A
4g8i	pMHC-I	-	-	C	A
4g9d	pMHC-I	-	-	C	A
4hux	pMHC-I	-	-	C	A
4jfp	pMHC-I	-	-	C	A
4jfp	pMHC-I	-	-	F	D
4nsk	pMHC-I	-	-	C	A
4pr5	pMHC-I	-	-	C	A
4pra	pMHC-I	-	-	C	A
4qrq	pMHC-I	-	-	C	A
4u1i	pMHC-I	-	-	C	A
4u1j	pMHC-I	-	-	C	A
4wu5	pMHC-I	-	-	C	A
4wu5	pMHC-I	-	-	F	D
4wu7	pMHC-I	-	-	C	A
4wu7	pMHC-I	-	-	F	D
5c0d	pMHC-I	-	-	C	A
5c0e	pMHC-I	-	-	C	A
5c0f	pMHC-I	-	-	C	A
5c0g	pMHC-I	-	-	C	A
5c0i	pMHC-I	-	-	C	A
5c0j	pMHC-I	-	-	C	A
5hga	pMHC-I	-	-	C	A
5hga	pMHC-I	-	-	F	D
5hgb	pMHC-I	-	-	C	A
5hgb	pMHC-I	-	-	F	D
5hgb	pMHC-I	-	-	I	G
5hgb	pMHC-I	-	-	L	J
5hgd	pMHC-I	-	-	C	A
5hgd	pMHC-I	-	-	F	D
5hgh	pMHC-I	-	-	C	A
5hhn	pMHC-I	-	-	C	A
5hhp	pMHC-I	-	-	C	A
5n1y	pMHC-I	-	-	C	A
5nmh	pMHC-I	-	-	C	A

Continued on next page

Table S1: Breakdown of the data used in the analysis of *apo* and *holo* forms of TCR and pMHC structures.

PDB ID	Structure Type	α-chain ID	β-chain ID	Antigen Chain ID	MHC Chain ID
5nmk	pMHC-I	-	-	C	A
5wjl	pMHC-I	-	-	C	A
5wjl	pMHC-I	-	-	F	D
5wjl	pMHC-I	-	-	I	G
5wjn	pMHC-I	-	-	C	A
5wjn	pMHC-I	-	-	F	D
5wjn	pMHC-I	-	-	I	G
5wli	pMHC-I	-	-	C	A
5wli	pMHC-I	-	-	F	D
5wli	pMHC-I	-	-	I	G
5wli	pMHC-I	-	-	L	J
5wmo	pMHC-I	-	-	C	A
5xos	pMHC-I	-	-	C	A
6amt	pMHC-I	-	-	C	A
6amt	pMHC-I	-	-	F	D
6at5	pMHC-I	-	-	C	A
6g9r	pMHC-I	-	-	I	C
6g9r	pMHC-I	-	-	J	E
6g9r	pMHC-I	-	-	K	G
6g9r	pMHC-I	-	-	P	A
6gh1	pMHC-I	-	-	P	A
6gh1	pMHC-I	-	-	Q	C
6gh1	pMHC-I	-	-	R	E
6gh1	pMHC-I	-	-	Z	G
6jtn	pMHC-I	-	-	C	A
6mt6	pMHC-I	-	-	B	A
6npr	pMHC-I	-	-	P	C
6npr	pMHC-I	-	-	R	A
6q3k	pMHC-I	-	-	P	A
6ulk	pMHC-I	-	-	C	A
6vr5	pMHC-I	-	-	P	A
6vr5	pMHC-I	-	-	Q	D
7mkb	pMHC-I	-	-	C	A
7n1a	pMHC-I	-	-	C	A
7n1a	pMHC-I	-	-	F	D
7n1b	pMHC-I	-	-	C	A
7n1b	pMHC-I	-	-	F	D
7n5q	pMHC-I	-	-	C	A
7n5q	pMHC-I	-	-	H	F
7n6d	pMHC-I	-	-	C	A
7n6d	pMHC-I	-	-	G	E
7n6d	pMHC-I	-	-	K	I
7n6d	pMHC-I	-	-	O	M
7n9j	pMHC-I	-	-	C	A
7nmd	pMHC-I	-	-	C	A
7nmd	pMHC-I	-	-	F	D
7ow3	pMHC-I	-	-	C	A
7ow3	pMHC-I	-	-	F	D

Continued on next page

Table S1: Breakdown of the data used in the analysis of *apo* and *holo* forms of TCR and pMHC structures.

PDB ID	Structure Type	α-chain ID	β-chain ID	Antigen Chain ID	MHC Chain ID
7ow3	pMHC-I	-	-	L	J
7ow4	pMHC-I	-	-	C	A
7ow4	pMHC-I	-	-	F	D
7ow4	pMHC-I	-	-	I	G
7p0a	pMHC-I	-	-	C	A
7p0a	pMHC-I	-	-	F	D
7p0t	pMHC-I	-	-	C	A
7p0t	pMHC-I	-	-	F	D
7p3d	pMHC-I	-	-	C	A
7r7v	pMHC-I	-	-	C	A
7rtd	pMHC-I	-	-	C	A
1kgc	TCR	D	E	-	-
1tcr	TCR	A	B	-	-
2bnu	TCR	A	B	-	-
2pyf	TCR	A	B	-	-
2vlm	TCR	D	E	-	-
3dx9	TCR	A	B	-	-
3dx9	TCR	C	D	-	-
3qeu	TCR	A	B	-	-
3qeu	TCR	D	E	-	-
3qh3	TCR	A	B	-	-
3skn	TCR	A	B	-	-
3skn	TCR	C	D	-	-
3skn	TCR	E	F	-	-
3skn	TCR	G	H	-	-
3utp	TCR	D	E	-	-
3utp	TCR	K	L	-	-
3vxq	TCR	A	B	-	-
3vxq	TCR	D	E	-	-
3vxt	TCR	A	B	-	-
3vxt	TCR	C	D	-	-
4jfh	TCR	D	E	-	-
4qrp	TCR	K	L	-	-
5iw1	TCR	A	B	-	-
5iw1	TCR	C	D	-	-
5iw1	TCR	E	F	-	-
5nmd	TCR	A	B	-	-
5nmd	TCR	C	D	-	-
6at6	TCR	A	B	-	-
6rp9	TCR	K	L	-	-
6vth	TCR	A	B	-	-
6vth	TCR	D	E	-	-
7amp	TCR	A	B	-	-
7n1c	TCR	D	E	-	-
7n1d	TCR	A	B	-	-
7r7z	TCR	A	B	-	-
1ao7	TCR:pMHC-I	D	E	C	A
1bd2	TCR:pMHC-I	D	E	C	A

Continued on next page

Table S1: Breakdown of the data used in the analysis of *apo* and *holo* forms of TCR and pMHC structures.

PDB ID	Structure Type	α-chain ID	β-chain ID	Antigen Chain ID	MHC Chain ID
1fo0	TCR:pMHC-I	A	B	P	H
1g6r	TCR:pMHC-I	A	B	P	H
1kj2	TCR:pMHC-I	A	B	P	H
1lp9	TCR:pMHC-I	E	F	C	A
1mi5	TCR:pMHC-I	D	E	C	A
1mwa	TCR:pMHC-I	A	B	P	H
1nam	TCR:pMHC-I	A	B	P	H
1oga	TCR:pMHC-I	D	E	C	A
1qrn	TCR:pMHC-I	D	E	C	A
1qse	TCR:pMHC-I	D	E	C	A
1qsf	TCR:pMHC-I	D	E	C	A
2ak4	TCR:pMHC-I	D	E	C	A
2bnq	TCR:pMHC-I	D	E	C	A
2bnr	TCR:pMHC-I	D	E	C	A
2ckb	TCR:pMHC-I	A	B	P	H
2f53	TCR:pMHC-I	D	E	C	A
2f54	TCR:pMHC-I	D	E	C	A
2j8u	TCR:pMHC-I	E	F	C	A
2jcc	TCR:pMHC-I	E	F	C	A
2ol3	TCR:pMHC-I	A	B	P	H
2p5e	TCR:pMHC-I	D	E	C	A
2p5w	TCR:pMHC-I	D	E	C	A
2pye	TCR:pMHC-I	D	E	C	A
2uwe	TCR:pMHC-I	E	F	C	A
2vlj	TCR:pMHC-I	D	E	C	A
2vlk	TCR:pMHC-I	D	E	C	A
2vlr	TCR:pMHC-I	D	E	C	A
3d39	TCR:pMHC-I	D	E	C	A
3d3v	TCR:pMHC-I	D	E	C	A
3dxa	TCR:pMHC-I	D	E	C	A
3ffc	TCR:pMHC-I	D	E	C	A
3gsn	TCR:pMHC-I	A	B	P	H
3h9s	TCR:pMHC-I	D	E	C	A
3hg1	TCR:pMHC-I	D	E	C	A
3kpr	TCR:pMHC-I	D	E	C	A
3kps	TCR:pMHC-I	D	E	C	A
3kxf	TCR:pMHC-I	D	E	Q	A
3o4l	TCR:pMHC-I	D	E	C	A
3pqy	TCR:pMHC-I	D	E	C	A
3pwp	TCR:pMHC-I	D	E	C	A
3qdg	TCR:pMHC-I	D	E	C	A
3qdj	TCR:pMHC-I	D	E	C	A
3qdm	TCR:pMHC-I	D	E	C	A
3qeq	TCR:pMHC-I	D	E	C	A
3qfj	TCR:pMHC-I	D	E	C	A
3sjv	TCR:pMHC-I	D	E	C	A
3uts	TCR:pMHC-I	D	E	C	A
3utt	TCR:pMHC-I	D	E	C	A

Continued on next page

Table S1: Breakdown of the data used in the analysis of *apo* and *holo* forms of TCR and pMHC structures.

PDB ID	Structure Type	α-chain ID	β-chain ID	Antigen Chain ID	MHC Chain ID
3vxm	TCR:pMHC-I	D	E	C	A
3vxr	TCR:pMHC-I	D	E	C	A
3vxs	TCR:pMHC-I	D	E	C	A
3vxu	TCR:pMHC-I	D	E	C	A
3w0w	TCR:pMHC-I	D	E	C	A
4eup	TCR:pMHC-I	G	H	F	D
4ftv	TCR:pMHC-I	D	E	C	A
4g8g	TCR:pMHC-I	D	E	C	A
4g9f	TCR:pMHC-I	D	E	C	A
4jfd	TCR:pMHC-I	D	E	C	A
4jfe	TCR:pMHC-I	D	E	C	A
4jff	TCR:pMHC-I	D	E	C	A
4jrx	TCR:pMHC-I	D	E	C	A
4jry	TCR:pMHC-I	D	E	C	A
4l3e	TCR:pMHC-I	D	E	C	A
4mji	TCR:pMHC-I	D	E	C	A
4prh	TCR:pMHC-I	D	E	C	A
4prp	TCR:pMHC-I	D	E	C	A
4qok	TCR:pMHC-I	D	E	C	A
4qrp	TCR:pMHC-I	D	E	C	A
5c07	TCR:pMHC-I	D	E	C	A
5c08	TCR:pMHC-I	D	E	C	A
5c09	TCR:pMHC-I	D	E	C	A
5c0a	TCR:pMHC-I	D	E	C	A
5c0b	TCR:pMHC-I	D	E	C	A
5c0c	TCR:pMHC-I	I	J	C	A
5d2n	TCR:pMHC-I	C	F	G	H
5e9d	TCR:pMHC-I	D	E	C	A
5euo	TCR:pMHC-I	E	F	J	A
5hhm	TCR:pMHC-I	D	E	C	A
5hho	TCR:pMHC-I	D	E	C	A
5hyj	TCR:pMHC-I	D	E	C	A
5isz	TCR:pMHC-I	D	E	C	A
5ivx	TCR:pMHC-I	E	F	P	A
5jhd	TCR:pMHC-I	D	E	C	A
5jzi	TCR:pMHC-I	D	E	C	A
5m00	TCR:pMHC-I	G	H	P	A
5m01	TCR:pMHC-I	G	H	P	A
5m02	TCR:pMHC-I	G	H	P	A
5nht	TCR:pMHC-I	A	B	P	H
5nme	TCR:pMHC-I	D	E	C	A
5nmf	TCR:pMHC-I	D	E	C	A
5nmg	TCR:pMHC-I	D	E	C	A
5nqk	TCR:pMHC-I	A	B	P	H
5sws	TCR:pMHC-I	D	E	C	A
5swz	TCR:pMHC-I	D	E	C	A
5til	TCR:pMHC-I	G	H	C	A
5tje	TCR:pMHC-I	G	H	I	A

Continued on next page

Table S1: Breakdown of the data used in the analysis of *apo* and *holo* forms of TCR and pMHC structures.

PDB ID	Structure Type	α-chain ID	β-chain ID	Antigen Chain ID	MHC Chain ID
5wkf	TCR:pMHC-I	D	E	C	A
5wkh	TCR:pMHC-I	D	E	C	A
5wlg	TCR:pMHC-I	D	E	C	A
5xot	TCR:pMHC-I	D	E	C	A
5xov	TCR:pMHC-I	I	J	C	A
5yxn	TCR:pMHC-I	A	B	I	C
6am5	TCR:pMHC-I	D	E	C	A
6amu	TCR:pMHC-I	D	E	C	A
6avf	TCR:pMHC-I	A	B	P	H
6avg	TCR:pMHC-I	C	D	P	G
6bj2	TCR:pMHC-I	D	E	C	A
6bj3	TCR:pMHC-I	D	H	C	A
6d78	TCR:pMHC-I	D	E	C	A
6dkp	TCR:pMHC-I	D	E	C	A
6eqa	TCR:pMHC-I	D	E	C	A
6eqb	TCR:pMHC-I	D	E	C	A
6g9q	TCR:pMHC-I	G	H	P	A
6mtm	TCR:pMHC-I	D	E	C	A
6q3s	TCR:pMHC-I	D	E	C	A
6rp9	TCR:pMHC-I	D	E	C	A
6rsy	TCR:pMHC-I	D	E	C	A
6tmo	TCR:pMHC-I	D	E	C	A
6tro	TCR:pMHC-I	D	E	C	A
6uon	TCR:pMHC-I	G	H	F	D
6uz1	TCR:pMHC-I	D	E	C	A
6vmx	TCR:pMHC-I	D	E	C	A
6vqo	TCR:pMHC-I	D	E	P	A
6vrm	TCR:pMHC-I	D	E	P	A
6vrn	TCR:pMHC-I	D	E	P	A
6zkw	TCR:pMHC-I	D	E	C	A
7dzm	TCR:pMHC-I	E	D	C	A
7dzn	TCR:pMHC-I	E	D	C	A
7jwi	TCR:pMHC-I	D	E	C	A
7jwj	TCR:pMHC-I	D	E	C	A
7n1e	TCR:pMHC-I	D	E	C	A
7n1f	TCR:pMHC-I	D	E	C	A
7n5c	TCR:pMHC-I	D	E	C	A
7n5p	TCR:pMHC-I	D	E	C	A
7n6e	TCR:pMHC-I	I	J	C	A
7na5	TCR:pMHC-I	D	E	C	A
7nme	TCR:pMHC-I	D	E	C	A
7nmf	TCR:pMHC-I	D	E	C	A
7ow5	TCR:pMHC-I	D	E	C	A
7ow6	TCR:pMHC-I	D	E	C	A
7pb2	TCR:pMHC-I	D	E	C	A
7pbe	TCR:pMHC-I	D	E	C	A
7r80	TCR:pMHC-I	A	B	E	C
7rm4	TCR:pMHC-I	E	D	C	A

Continued on next page

Table S1: Breakdown of the data used in the analysis of *apo* and *holo* forms of TCR and pMHC structures.

PDB ID	Structure Type	α-chain ID	β-chain ID	Antigen Chain ID	MHC Chain ID
7rtr	TCR:pMHC-I	D	E	C	A
8gvb	TCR:pMHC-I	A	B	P	H
8gvg	TCR:pMHC-I	A	B	P	H
8gvi	TCR:pMHC-I	A	B	P	H

Table S2: TCR comparisons made in the analysis. For each TCR, pair-wise combinations of all structures are computed. All results in the paper are normalised by taking the mean of the measurement per TCR so that TCRs with higher numbers of structures do not bias the results. TCRs are denoted by the sequence of all their CDR loops.

State	PDB ID	α-chain ID	β-chain ID	Antigen Chain ID	MHC Chain ID
...ATGYPS...ATKADDK...ALSDPVNDMR..., ...SGHAT...FQNNGV...ASSLRGRGDQPQH...					
apo	4qrp	K	L	-	-
holo	4qrp	D	E	C	A
...DRGSQS...IYSNGD...ALTRGPGNQFY..., ...SGHVS...FNYEAQ...ASSSPGGVSTEAF...					
apo	6rp9	K	L	-	-
holo	6rp9	D	E	C	A
...DRGSQS...IYSNGD...AVNFGGGKLI..., ...MRHNA...SNTAGT...ASSLSFGTEAF...					
apo	3qeu	A	B	-	-
apo	3qeu	D	E	-	-
holo	3qdg	D	E	C	A
holo	3qdj	D	E	C	A
holo	6am5	D	E	C	A
holo	6amu	D	E	C	A
...DRGSQS...IYSNGD...AVNRDDKII..., ...SEHNR...FQNEAQ...ASSPDIEQY...					
apo	7n1d	A	B	-	-
holo	7n1f	D	E	C	A
holo	7rtr	D	E	C	A
...DRGSQS...IYSNGD...AVNVAGKST..., ...GTSNPN...SVGIG...AWSETGLGTGELF...					
holo	3hg1	D	E	C	A
holo	4qok	D	E	C	A
holo	6eqa	D	E	C	A
...DRGSQS...IYSNGD...AVRTNSGYALN..., ...QGHDT...YYEEEE...ASSDTVSYEQY...					
apo	5nmd	A	B	-	-
apo	5nmd	C	D	-	-
holo	5nme	D	E	C	A
holo	5nmf	D	E	C	A
holo	5nmg	D	E	C	A
...DRGSQS...IYSNGD...AVTTDSWGKLQ..., ...MNHEY...SVGAGI...ASRPGLAGGRPEQY...					
apo	3qh3	A	B	-	-
apo	7amp	A	B	-	-
holo	1ao7	D	E	C	A
holo	1qrn	D	E	C	A
holo	1qse	D	E	C	A
holo	1qsf	D	E	C	A
holo	3d39	D	E	C	A

Continued on next page

Table S2: TCR comparisons made in the analysis.

State	PDB ID	α-chain ID	β-chain ID	Antigen Chain ID	MHC Chain ID
holo	3d3v	D	E	C	A
holo	3h9s	D	E	C	A
holo	3pwp	D	E	C	A
holo	3qfj	D	E	C	A
...DRGSQS...IYSNGD...GTYNQGGKLI..., ...MNHEY...SMNVEV...ASSGASHEQY...					
apo	3vxt	A	B	-	-
apo	3vxt	C	D	-	-
holo	3vxu	D	E	C	A
holo	3w0w	D	E	C	A
...DSAIYN...IQSSQRE...AQLNQAGTALI..., ...MNHEY...SVGAGI...ASSYGTGINYGYT...					
apo	7r7z	A	B	-	-
holo	7r80	A	B	E	C
...DSAIYN...IQSSQRE...AVRMDSSYKLI..., ...SEHNR...FQNEAQ...ASSSWDTGELF...					
apo	3vxq	A	B	-	-
apo	3vxq	D	E	-	-
holo	3vxr	D	E	C	A
holo	3vxs	D	E	C	A
...DSAIYN...IQSSQRE...AVRPLLDGTYIPT..., ...MNHEY...SVGAGT...ASSYLGNTGELF...					
apo	2pyf	A	B	-	-
holo	2pye	D	E	C	A
...DSAIYN...IQSSQRE...AVRPTSGGSYIPT..., ...MNHEY...SVGAGI...ASSYVGNTGELF...					
apo	2bnu	A	B	-	-
holo	2bnq	D	E	C	A
holo	2bnr	D	E	C	A
holo	2f54	D	E	C	A
holo	6q3s	D	E	C	A
...DSSSTY...IFSNMDM...AEPSGNTGKLI..., ...SEHNR...FQNEAQ...ASSLHHEQY...					
holo	7nme	D	E	C	A
holo	7nmf	D	E	C	A
...DSTFNY...ILSVSDK...AALYGNEKIT..., ...NNHDY...SYVADS...ASSDAGGRNTLY...					
holo	5m00	G	H	P	A
holo	5m01	G	H	P	A
holo	5m02	G	H	P	A
holo	5til	G	H	C	A
holo	5tje	G	H	I	A
holo	6g9q	G	H	P	A
...DSTFNY...IRSVSDK...AASETSGSWQLI..., ...MNHDT...YYDKIL...ASSRDLGRDTQY...					
holo	5swz	D	E	C	A
holo	7jwi	D	E	C	A
...FLGSQS...TYREGD...AVNDGGRLT..., ...GTSNPN...WGPFG...AWSETGLGMGGWQ...					
apo	4jfh	D	E	-	-
holo	4jfd	D	E	C	A
holo	4jfe	D	E	C	A
holo	4jff	D	E	C	A
holo	6eqb	D	E	C	A
holo	6tmo	D	E	C	A
...KALYS...LLKGGEQ...GLGDAGNMLT..., ...SGHAT...FQNNGV...ASSLGQGLLYGYT...					
holo	5wkf	D	E	C	A

Continued on next page

Table S2: TCR comparisons made in the analysis.

State	PDB ID	α-chain ID	β-chain ID	Antigen Chain ID	MHC Chain ID
holo	5wkh	D	E	C	A
...NIATNDY...GYKTK...LVGEILDNFNKFY..., ...MDHEN...SYDVKM...ASSQRQEGDTQY...					
apo	6at6	A	B	-	-
holo	6avf	A	B	P	H
...NSAFDY...ILSVSNK...AASASFGDNSKLI..., ...MSHET...SYDVDS...ASSLGHTEVF...					
apo	5iw1	A	B	-	-
apo	5iw1	C	D	-	-
apo	5iw1	E	F	-	-
holo	5ivx	E	F	P	A
...NSAFQY...TYSSGN...AMRGDSSYKLI..., ...SGHDY...FNNNVP...ASSLWEKLAKNIQY...					
apo	3utp	D	E	-	-
apo	3utp	K	L	-	-
holo	3uts	D	E	C	A
holo	3utt	D	E	C	A
holo	5c07	D	E	C	A
holo	5c08	D	E	C	A
holo	5c09	D	E	C	A
holo	5c0a	D	E	C	A
holo	5c0b	D	E	C	A
holo	5c0c	I	J	C	A
holo	5hyj	D	E	C	A
...NSASQS...VYSSG...VVQPGGYQKVT..., ...MNHNS...SASEGT...ASSEGLWQVGDEQY...					
apo	6vth	A	B	-	-
apo	6vth	D	E	-	-
holo	6vrm	D	E	P	A
...NSASQS...VYSSG...VVRAGKLI..., ...MNHEY...SVGEGT...ASGQGNFDIQY...					
apo	3skn	A	B	-	-
apo	3skn	C	D	-	-
apo	3skn	E	F	-	-
apo	3skn	G	H	-	-
holo	3sjv	D	E	C	A
...SIFNT...LYKAGEL...AGGTGNQFY..., ...ENHRY...SYGVKD...AISEVGVGQPQH...					
holo	3qdm	D	E	C	A
holo	3qeq	D	E	C	A
...STYSPF...SFTDNKR...ALFLASSSFSKLV..., ...NNHDY...SYVADS...ASSDWVSYEQY...					
holo	1lp9	E	F	C	A
holo	2j8u	E	F	C	A
holo	2jcc	E	F	C	A
holo	2uwe	E	F	C	A
...SVFSS...VVTGGEV...AGAGSQGNLI..., ...LNHDA...SQIVND...ASSSRSSYEQY...					
apo	2vlm	D	E	-	-
holo	1oga	D	E	C	A
holo	2vlj	D	E	C	A
holo	2vlk	D	E	C	A
holo	5hhm	D	E	C	A
...TISGNEY...GLKNN...IVRGLNNAGNMLT..., ...SGHNS...FNNNVP...ASSLGIDAIY...					
holo	7dzm	E	D	C	A
holo	7dzn	E	D	C	A

Continued on next page

Table S2: TCR comparisons made in the analysis.

State	PDB ID	α-chain ID	β-chain ID	Antigen Chain ID	MHC Chain ID
...TISGNEY...GLKNN...IVWGGYQKVT..., ...SEHNR...FQNEAQ...ASRYRDDSYNEQF...					
apo	3dx9	A	B	-	-
apo	3dx9	C	D	-	-
holo	3dxa	D	E	C	A
...TISGNEY...GLQQN...ILSGGSNYKLT..., ...MSHET...SYDVDS...ASSFGREQY...					
holo	3pqy	D	E	C	A
holo	7n5p	D	E	C	A
...TISGTDY...GLTSN...ILPLAGGTSYGKLT..., ...SGHVS...FQNEAQ...ASSLGQAYEQY...					
apo	1kgc	D	E	-	-
holo	1mi5	D	E	C	A
holo	3kpr	D	E	C	A
holo	3kps	D	E	C	A
...TQDSSYF...QDSYKKEN...AMRGDYGGSGNKLI..., ...NSQYPW...LRSPGD...TCSADRVGNTLY...					
holo	1fo0	A	B	P	H
holo	1nam	A	B	P	H
holo	2ol3	A	B	P	H
...TRDTAYY...QPWWGEQN...AMSVPSGDGSYQFT..., ...MNHEY...SVGEGT...ASKVGPGQHNSPLH...					
holo	7ow5	D	E	C	A
holo	7ow6	D	E	C	A
...TRDTTYY...RNSFDEQN...ALSGFYNTDKLI..., ...MNHNS...SASEGT...ASPGLAGEYEQY...					
holo	2ak4	D	E	C	A
holo	3kxf	D	E	Q	A
...TSDQSYG...QGSYDEQN...AMRDLRDNFNKFY..., ...MNHEY...SVGAGI...ASREGLGGTEAF...					
holo	4g8g	D	E	C	A
holo	4g9f	D	E	C	A
...TSESDYY...QEAYKQQN...AYGEDDKII..., ...MGHDK...SYGVNS...ASRRGPYEQY...					
holo	5jzi	D	E	C	A
holo	5yxn	A	B	I	C
...TSWWSYY...QGSDEQN...ALGEGGAQKLV..., ...MNHNS...SASEGT...ASRTRGGTLIEQY...					
holo	5xot	D	E	C	A
holo	6bj3	D	H	C	A
...VSGLRG...LYSAGEE...AVQDLGTSGSRLT..., ...SGDLS...YYNGEE...ASSARSGELF...					
holo	4prh	D	E	C	A
holo	4prp	D	E	C	A
...YGATPY...YFSGDTLV...AVGFTGGGNKLT..., ...SEHNR...FQNEAQ...ASSDRDRVPETQY...					
holo	8gvb	A	B	P	H
holo	8gvg	A	B	P	H
...YRGSQS...IYSNGD...AVNFGGGKLI..., ...MRHNA...SNTAGT...ASSWSFGTEAF...					
holo	4l3e	D	E	C	A
holo	6d78	D	E	C	A
...YSATPY...YYSGDPVV...AVSGFASALT..., ...NNHNN...SYGAGS...ASGGGGTLY...					
apo	1tcr	A	B	-	-
holo	1g6r	A	B	P	H
holo	1mwa	A	B	P	H
holo	2ckb	A	B	P	H
...YSGSPE...HISR...ALSGFNNAGNMLT..., ...SGHAT...FQNNGV...ASSLGGAGGADTQY...					
apo	7n1c	D	E	-	-
holo	7n1e	D	E	C	A

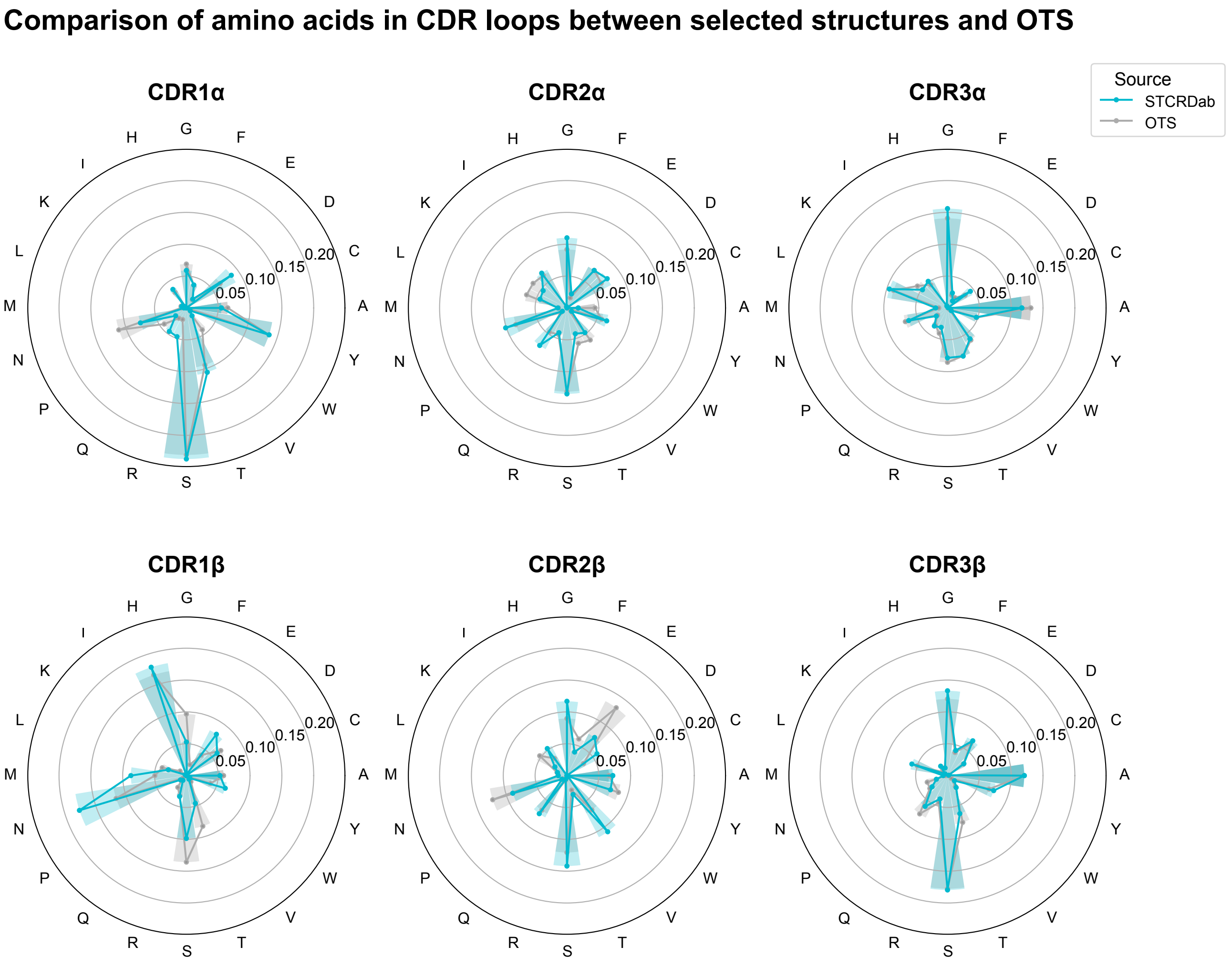

Figure S1. Comparison of the composition of amino acid residues in each CDR loop between the structures in this analysis and a comparative background of TCRs randomly sampled from Observed TCR Space (OTS) Raybould et al. (2024).

Table S3: pMHC-I comparisons made in the analysis. For each pMHC-I, pair-wise combinations of all structures are computed. All results in the paper are normalised by taking the mean of the measurement per pMHC-I so that pMHC-Is with higher numbers of structures do not bias the results. The pMHC-Is are denoted by their peptide sequence and allele code.

State	PDB ID	α-chain ID	β-chain ID	Antigen Chain ID	MHC Chain ID
ASNENMETM, H2-Db					
apo	1hoc	-	-	C	A
apo	4hux	-	-	C	A
holo	5sws	D	E	C	A
holo	5swz	D	E	C	A
holo	7jwi	D	E	C	A
holo	7jwj	D	E	C	A

Continued on next page

Table S3: pMHC-I comparisons made in the analysis.

State	PDB ID	α-chain ID	β-chain ID	Antigen Chain ID	MHC Chain ID
KAPANFATM, H2-Db					
apo	3tbs	-	-	C	A
apo	3tbs	-	-	F	D
holo	5m01	G	H	P	A
KAPFNFATM, H2-Db					
apo	3tby	-	-	C	A
apo	3tby	-	-	F	D
apo	3tby	-	-	I	G
apo	3tby	-	-	L	J
holo	5m02	G	H	P	A
KAPYDYAPI, H2-Db					
apo	6g9r	-	-	I	C
apo	6g9r	-	-	J	E
apo	6g9r	-	-	K	G
apo	6g9r	-	-	P	A
holo	6g9q	G	H	P	A
KAPYNFATM, H2-Db					
apo	4nsk	-	-	C	A
apo	7p0a	-	-	C	A
apo	7p0a	-	-	F	D
holo	5til	G	H	C	A
KAVANFATM, H2-Db					
apo	3quk	-	-	C	A
apo	3quk	-	-	F	D
holo	5m00	G	H	P	A
KAVYNFATM, H2-Db					
apo	1ffn	-	-	C	A
apo	1ffn	-	-	F	D
apo	1n5a	-	-	C	A
apo	1n5a	-	-	F	D
apo	1n5a	-	-	I	G
apo	1n5a	-	-	L	J
apo	1s7u	-	-	C	A
apo	1s7u	-	-	F	D
apo	1s7u	-	-	I	G
apo	1s7u	-	-	L	J
apo	2f74	-	-	C	A
apo	2f74	-	-	F	D
apo	7p0t	-	-	C	A
apo	7p0t	-	-	F	D
holo	5tje	G	H	I	A
SQLLNAKYL, H2-Db					
apo	5wli	-	-	C	A
apo	5wli	-	-	F	D
apo	5wli	-	-	I	G
apo	5wli	-	-	L	J
holo	5wlg	D	E	C	A
SSLCNFRAYV, H2-Db					

Continued on next page

Table S3: pMHC-I comparisons made in the analysis.

State	PDB ID	α-chain ID	β-chain ID	Antigen Chain ID	MHC Chain ID
apo	7n5q	-	-	C	A
apo	7n5q	-	-	H	F
holo	7n5c	D	E	C	A
holo	7n5p	D	E	C	A
SSLENFRAYV, H2-Db					
apo	1wby	-	-	C	A
apo	1yn6	-	-	C	A
holo	3pqy	D	E	C	A
YGFRNVVHI, H2-Db					
apo	7n9j	-	-	C	A
holo	7na5	D	E	C	A
RGPGRAFVTI, H2-Dd					
apo	1bii	-	-	P	A
apo	1ddh	-	-	P	A
apo	3ecb	-	-	P	A
apo	6npr	-	-	P	C
apo	6npr	-	-	R	A
holo	5ivx	E	F	P	A
EQYKFYSV, H2-Kb					
apo	1leg	-	-	P	A
apo	1lek	-	-	P	A
holo	1mwa	A	B	P	H
holo	2ckb	A	B	P	H
INFDFNTI, H2-Kb					
apo	1nan	-	-	M	H
apo	2clz	-	-	C	A
apo	2clz	-	-	M	H
holo	1fo0	A	B	P	H
KVITFIDL, H2-Kb					
apo	1kj3	-	-	P	H
apo	1kj3	-	-	Q	I
holo	1kj2	A	B	P	H
RGYVYQGL, H2-Kb					
apo	1fzj	-	-	P	A
apo	1fzm	-	-	P	A
apo	1kpu	-	-	P	A
apo	2mha	-	-	E	A
apo	2mha	-	-	F	C
apo	2vaa	-	-	P	A
holo	1nam	A	B	P	H
SQYYYNSL, H2-Kb					
apo	2clv	-	-	C	A
apo	2clv	-	-	M	H
holo	2ol3	A	B	P	H
AAGIGILTV, HLA-A*02:01					
apo	2guo	-	-	C	A
apo	2guo	-	-	F	D
apo	3qfd	-	-	C	A

Continued on next page

Table S3: pMHC-I comparisons made in the analysis.

State	PDB ID	α-chain ID	β-chain ID	Antigen Chain ID	MHC Chain ID
apo	3qfd	-	-	F	D
holo	3qdj	D	E	C	A
holo	3qeq	D	E	C	A
holo	6d78	D	E	C	A
holo	6eqa	D	E	C	A
holo	6eqb	D	E	C	A
ALGIGILTV, HLA-A*02:01					
apo	1jht	-	-	C	A
apo	2gtz	-	-	C	A
apo	2gtz	-	-	F	D
holo	4eup	G	H	F	D
ALWGFFPVL, HLA-A*02:01					
apo	1b0g	-	-	C	A
apo	1b0g	-	-	F	D
holo	1lp9	E	F	C	A
holo	2j8u	E	F	C	A
holo	2jcc	E	F	C	A
holo	2uwe	E	F	C	A
ALWGPDPAAA, HLA-A*02:01					
apo	3utq	-	-	C	A
holo	3uts	D	E	C	A
holo	3utt	D	E	C	A
AQWGPDPAAA, HLA-A*02:01					
apo	5c0d	-	-	C	A
holo	5hyj	D	E	C	A
EAAGIGILTV, HLA-A*02:01					
apo	2gt9	-	-	C	A
apo	2gt9	-	-	F	D
holo	4qok	D	E	C	A
holo	6tmo	D	E	C	A
ELAAIGILTV, HLA-A*02:01					
apo	4jfp	-	-	C	A
apo	4jfp	-	-	F	D
holo	4jfd	D	E	C	A
ELAGIGILTV, HLA-A*02:01					
apo	1jf1	-	-	C	A
holo	3hg1	D	E	C	A
holo	3qdg	D	E	C	A
holo	3qdm	D	E	C	A
holo	4jff	D	E	C	A
holo	4l3e	D	E	C	A
holo	5e9d	D	E	C	A
holo	5nht	A	B	P	H
holo	5nqk	A	B	P	H
holo	6dkp	D	E	C	A
GILEFVFTL, HLA-A*02:01					
apo	5hhp	-	-	C	A
holo	5hho	D	E	C	A

Continued on next page

Table S3: pMHC-I comparisons made in the analysis.

State	PDB ID	α-chain ID	β-chain ID	Antigen Chain ID	MHC Chain ID
GILGFVFTL, HLA-A*02:01					
apo	1hhi	-	-	C	A
apo	1hhi	-	-	F	D
apo	2vll	-	-	C	A
apo	2vll	-	-	F	D
holo	1oga	D	E	C	A
holo	2vlj	D	E	C	A
holo	2vlk	D	E	C	A
holo	2vlr	D	E	C	A
holo	5euo	E	F	J	A
holo	5isz	D	E	C	A
holo	5jhd	D	E	C	A
GILGLVFTL, HLA-A*02:01					
apo	5hhn	-	-	C	A
holo	5hhm	D	E	C	A
GLCTLVAML, HLA-A*02:01					
apo	3mre	-	-	P	A
holo	3o4l	D	E	C	A
GVYDGREHTV, HLA-A*02:01					
apo	1i4f	-	-	C	A
holo	6tro	D	E	C	A
HMTEVVRHC, HLA-A*02:01					
apo	6vr5	-	-	P	A
apo	6vr5	-	-	Q	D
holo	6vqo	D	E	P	A
holo	6vrm	D	E	P	A
holo	6vrn	D	E	P	A
holo	7rm4	E	D	C	A
KLVALGINAV, HLA-A*02:01					
apo	3mrm	-	-	P	A
holo	5jzi	D	E	C	A
holo	5yxn	A	B	I	C
LGYGFVNYI, HLA-A*02:01					
apo	3pwl	-	-	C	A
apo	3pwl	-	-	F	D
holo	3pwp	D	E	C	A
LLFGFPVYV, HLA-A*02:01					
holo	3d39	D	E	C	A
holo	3d3v	D	E	C	A
holo	3qfj	D	E	C	A
LLFGYPVYV, HLA-A*02:01					
apo	1duz	-	-	C	A
apo	1duz	-	-	F	D
apo	1hhk	-	-	C	A
apo	1hhk	-	-	F	D
apo	2av1	-	-	C	A
apo	2av1	-	-	F	D
apo	2av7	-	-	C	A

Continued on next page

Table S3: pMHC-I comparisons made in the analysis.

State	PDB ID	α-chain ID	β-chain ID	Antigen Chain ID	MHC Chain ID
apo	2av7	-	-	F	D
apo	3ixa	-	-	C	A
apo	3ixa	-	-	F	D
holo	1ao7	D	E	C	A
holo	1bd2	D	E	C	A
holo	4ftv	D	E	C	A
holo	6uz1	D	E	C	A
MLWGYLQYV, HLA-A*02:01					
apo	3h7b	-	-	C	A
apo	3h7b	-	-	F	D
apo	3h9h	-	-	C	A
apo	3h9h	-	-	F	D
holo	3h9s	D	E	C	A
MMWDRGLGMM, HLA-A*02:01					
apo	6amt	-	-	C	A
apo	6amt	-	-	F	D
holo	6amu	D	E	C	A
MVWGPDPLYV, HLA-A*02:01					
apo	5n1y	-	-	C	A
holo	5c0a	D	E	C	A
NLVPMVATV, HLA-A*02:01					
apo	2x4r	-	-	C	A
apo	2x4r	-	-	F	D
apo	3gso	-	-	P	A
apo	6q3k	-	-	P	A
holo	3gsn	A	B	P	H
holo	5d2n	C	F	G	H
RLQSLQTYV, HLA-A*02:01					
apo	7n1b	-	-	C	A
apo	7n1b	-	-	F	D
holo	7n1e	D	E	C	A
RMFPNAPYL, HLA-A*02:01					
apo	3hpj	-	-	C	A
apo	3hpj	-	-	F	D
holo	6rsy	D	E	C	A
RQFGPDFPTI, HLA-A*02:01					
apo	5c0i	-	-	C	A
holo	5c0b	D	E	C	A
RQFGPDWIVA, HLA-A*02:01					
apo	5c0j	-	-	C	A
holo	5c0c	I	J	C	A
RQWGPDPAAV, HLA-A*02:01					
apo	5c0f	-	-	C	A
holo	5c08	D	E	C	A
SLFNTIAVL, HLA-A*02:01					
apo	1t1w	-	-	C	A
apo	2c7u	-	-	C	A
apo	2c7u	-	-	F	D

Continued on next page

Table S3: pMHC-I comparisons made in the analysis.

State	PDB ID	α-chain ID	β-chain ID	Antigen Chain ID	MHC Chain ID
apo	5nmk	-	-	C	A
holo	5nmg	D	E	C	A
SLLMWITQC, HLA-A*02:01					
apo	1s9w	-	-	C	A
holo	2bnr	D	E	C	A
holo	2f53	D	E	C	A
holo	2f54	D	E	C	A
holo	2p5e	D	E	C	A
holo	2p5w	D	E	C	A
holo	2pye	D	E	C	A
SLLMWITQV, HLA-A*02:01					
holo	2bnq	D	E	C	A
holo	6q3s	D	E	C	A
holo	6rp9	D	E	C	A
SLYNTIATL, HLA-A*02:01					
apo	1t20	-	-	C	A
apo	5nmh	-	-	C	A
holo	5nmf	D	E	C	A
SLYNTVATL, HLA-A*02:01					
apo	1t21	-	-	C	A
apo	1t22	-	-	C	A
apo	2v2w	-	-	C	A
apo	2v2w	-	-	F	D
holo	5nme	D	E	C	A
YLGGPDFPTI, HLA-A*02:01					
apo	5c0g	-	-	C	A
holo	5c09	D	E	C	A
YLQPRTFLL, HLA-A*02:01					
apo	7mkb	-	-	C	A
apo	7n1a	-	-	C	A
apo	7n1a	-	-	F	D
apo	7n6d	-	-	C	A
apo	7n6d	-	-	G	E
apo	7n6d	-	-	K	I
apo	7n6d	-	-	O	M
apo	7p3d	-	-	C	A
apo	7rtd	-	-	C	A
holo	7n1f	D	E	C	A
holo	7n6e	I	J	C	A
holo	7pbe	D	E	C	A
holo	7rtr	D	E	C	A
YQFGPDFPIA, HLA-A*02:01					
apo	5c0e	-	-	C	A
holo	5c07	D	E	C	A
GTSGSPIINR, HLA-A*11:01					
apo	5wjn	-	-	C	A
apo	5wjn	-	-	F	D
apo	5wjn	-	-	I	G

Continued on next page

Table S3: pMHC-I comparisons made in the analysis.

State	PDB ID	α-chain ID	β-chain ID	Antigen Chain ID	MHC Chain ID
holo	5wkh	D	E	C	A
GTSGSPIVNR, HLA-A*11:01					
apo	5wjl	-	-	C	A
apo	5wjl	-	-	F	D
apo	5wjl	-	-	I	G
holo	5wkf	D	E	C	A
VVVGADGVGK, HLA-A*11:01					
apo	7ow4	-	-	C	A
apo	7ow4	-	-	F	D
apo	7ow4	-	-	I	G
holo	7ow6	D	E	C	A
holo	7pb2	D	E	C	A
VVVGAGGVGK, HLA-A*11:01					
apo	7ow3	-	-	C	A
apo	7ow3	-	-	F	D
apo	7ow3	-	-	L	J
holo	7ow5	D	E	C	A
QLPRLFPLL, HLA-A*24:02					
apo	7nmd	-	-	C	A
apo	7nmd	-	-	F	D
holo	7nme	D	E	C	A
holo	7nmf	D	E	C	A
RFPLTFGW, HLA-A*24:02					
apo	4wu7	-	-	C	A
apo	4wu7	-	-	F	D
apo	5hga	-	-	C	A
apo	5hga	-	-	F	D
holo	8gvg	A	B	P	H
RFPLTFGWCF, HLA-A*24:02					
apo	3vxo	-	-	C	A
apo	3vxo	-	-	F	D
apo	5hgd	-	-	C	A
apo	5hgd	-	-	F	D
holo	3vxm	D	E	C	A
holo	3vxu	D	E	C	A
holo	3w0w	D	E	C	A
RYPLTFGW, HLA-A*24:02					
apo	4wu5	-	-	C	A
apo	4wu5	-	-	F	D
apo	5hgb	-	-	C	A
apo	5hgb	-	-	F	D
apo	5hgb	-	-	I	G
apo	5hgb	-	-	L	J
holo	8gvb	A	B	P	H
holo	8gvi	A	B	P	H
RYPLTFGWCF, HLA-A*24:02					
apo	3nfn	-	-	C	A
apo	3vxn	-	-	C	A

Continued on next page

Table S3: pMHC-I comparisons made in the analysis.

State	PDB ID	α-chain ID	β-chain ID	Antigen Chain ID	MHC Chain ID
apo	5hgh	-	-	C	A
holo	3vxr	D	E	C	A
holo	5xov	I	J	C	A
RYPLTLGWCF, HLA-A*24:02					
apo	3vxp	-	-	C	A
apo	3vxp	-	-	F	D
holo	3vxs	D	E	C	A
APRGPHGGAASGL, HLA-B*07:02					
apo	6at5	-	-	C	A
holo	6avf	A	B	P	H
holo	6avg	C	D	P	G
RPPIFIRRL, HLA-B*07:02					
apo	5wmo	-	-	C	A
holo	6vmx	D	E	C	A
FLRGRAYGL, HLA-B*08:01					
apo	1m05	-	-	E	A
apo	1m05	-	-	F	C
apo	3sko	-	-	C	A
apo	3x13	-	-	C	A
holo	1mi5	D	E	C	A
holo	3ffc	D	E	C	A
holo	3sjv	D	E	C	A
HSKKKCDEL, HLA-B*08:01					
apo	4qrq	-	-	C	A
holo	4qrp	D	E	C	A
KRWIILGLNK, HLA-B*27:05					
apo	2bss	-	-	C	A
apo	4g9d	-	-	C	A
holo	4g8g	D	E	C	A
KRWIIMGLNK, HLA-B*27:05					
apo	4g8i	-	-	C	A
holo	4g9f	D	E	C	A
HPVGDADYFEY, HLA-B*35:01					
apo	4pr5	-	-	C	A
holo	4prh	D	E	C	A
HPVGQADYFEY, HLA-B*35:01					
apo	4pra	-	-	C	A
holo	4prp	D	E	C	A
IPLTEEAEL, HLA-B*35:01					
apo	5xos	-	-	C	A
holo	5xot	D	E	C	A
holo	6bj2	D	E	C	A
holo	6bj3	D	H	C	A
LPEPLPQGQLTAY, HLA-B*35:01					
apo	1zhk	-	-	C	A
apo	3kww	-	-	C	A
apo	3vfo	-	-	C	A
holo	3kxf	D	E	Q	A

Continued on next page

Table S3: pMHC-I comparisons made in the analysis.

State	PDB ID	α-chain ID	β-chain ID	Antigen Chain ID	MHC Chain ID
LPEPLPQGQLTAY, HLA-B*35:08					
apo	1zhl	-	-	C	A
apo	3vfm	-	-	C	A
apo	3vfn	-	-	C	A
apo	3vfp	-	-	C	A
holo	2ak4	D	E	C	A
holo	4jrx	D	E	C	A
holo	4jry	D	E	C	A
FEDLRVLSF, HLA-B*37:01					
apo	6mt6	-	-	B	A
holo	6mtm	D	E	C	A
TPQDLNTML, HLA-B*42:01					
apo	4u1j	-	-	C	A
holo	7dzn	E	D	C	A
EENLLDFVRF, HLA-B*44:05					
apo	3dx8	-	-	C	A
holo	3dxa	D	E	C	A
EEYLKAWTF, HLA-B*44:05					
apo	3kpq	-	-	C	A
holo	3kpr	D	E	C	A
EEYLQAFTY, HLA-B*44:05					
apo	3kpp	-	-	C	A
holo	3kps	D	E	C	A
TAFTIPSI, HLA-B*51:01					
apo	1e28	-	-	C	A
holo	4mji	D	E	C	A
QASQEVKNW, HLA-B*53:01					
apo	7r7v	-	-	C	A
holo	7r80	A	B	E	C
TPQDLNTML, HLA-B*81:01					
apo	4u1i	-	-	C	A
holo	7dzm	E	D	C	A
GADGVGKSAL, HLA-C*08:02					
apo	6jtn	-	-	C	A
apo	6ulk	-	-	C	A
holo	6uon	G	H	F	D
RLPAKAPLL, HLA-E*01:03					
apo	6gh1	-	-	P	A
apo	6gh1	-	-	Q	C
apo	6gh1	-	-	R	E
apo	6gh1	-	-	Z	G
holo	6zkw	D	E	C	A

REFERENCES

Borrman T, Cimons J, Cosiano M, Purcaro M, Pierce BG, Baker BM, et al. ATLAS: A database linking binding affinities with structures for wild-type and mutant TCR-pMHC complexes. *Proteins: Structure, Function, and Bioinformatics* **85** (2017) 908–916. doi:10.1002/prot.25260.

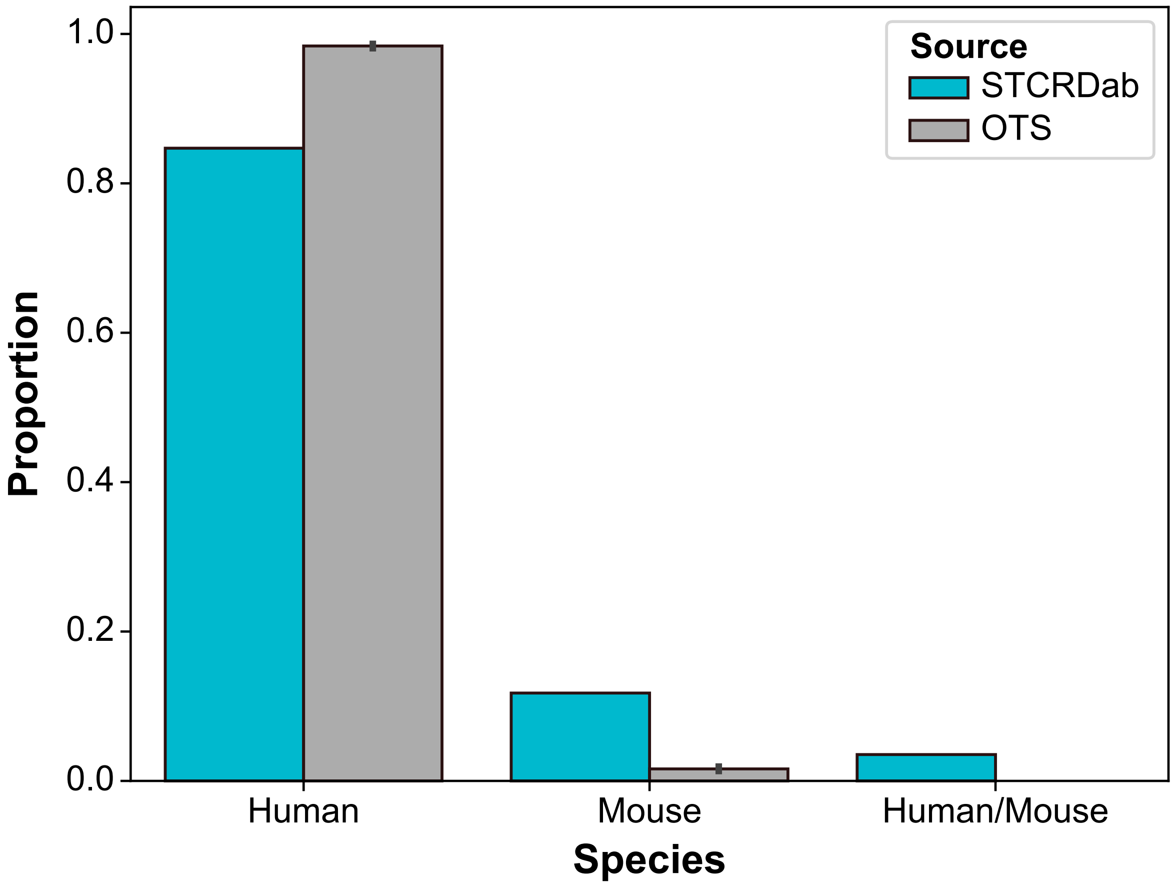

Figure S2. Proportion of the species of origin for the TCRs in the selected STCRDab structures and the randomly sampled OTS background.

Milighetti M, Nagano Y, Henderson J, Hershberg U, Tiffeau-Mayer A, Bitbol AF, et al. Intra- and inter-chain contacts determine TCR specificity: Applying protein co-evolution methods to TCRαβ pairing (2024). doi:10.1101/2024.05.24.595718.

Raybould MI, Greenshields-Watson A, Agarwal P, Aguilar-Sanjuan B, Olsen TH, Turnbull OM, et al. The observed t cell receptor space database enables paired-chain repertoire mining, coherence analysis and language modelling. *bioRxiv* (2024). doi:10.1101/2024.05.20.594960.

Leem J, de Oliveira SH, Krawczyk K, Deane CM. STCRDab: the structural t-cell receptor database. *Molecular Cell* **46** (2018) D406–D412. doi:10.1093/nar/gkx971.

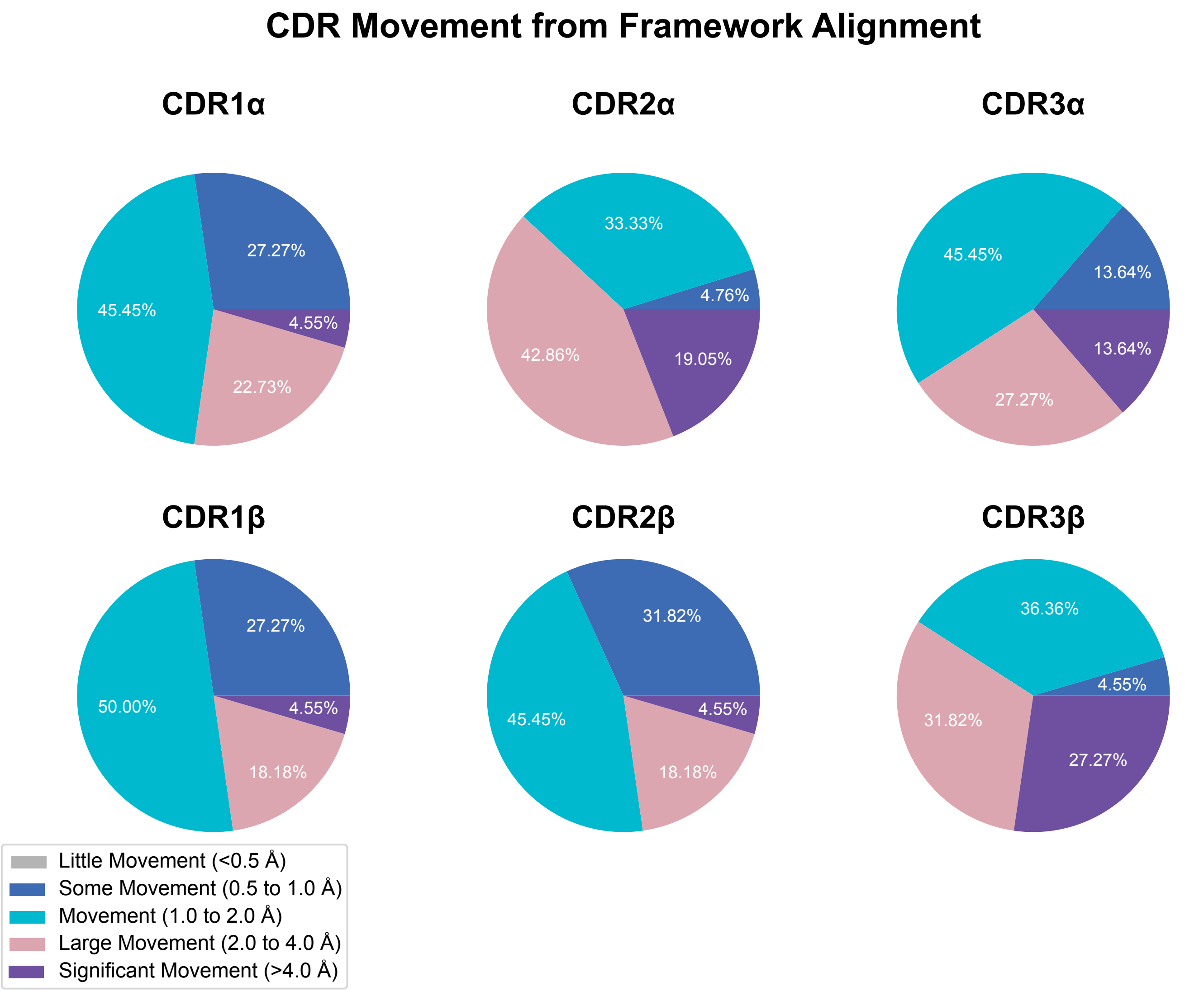

Figure S3. Percentage of different movement categories from ech CDR type after alignment on TCR framework region.

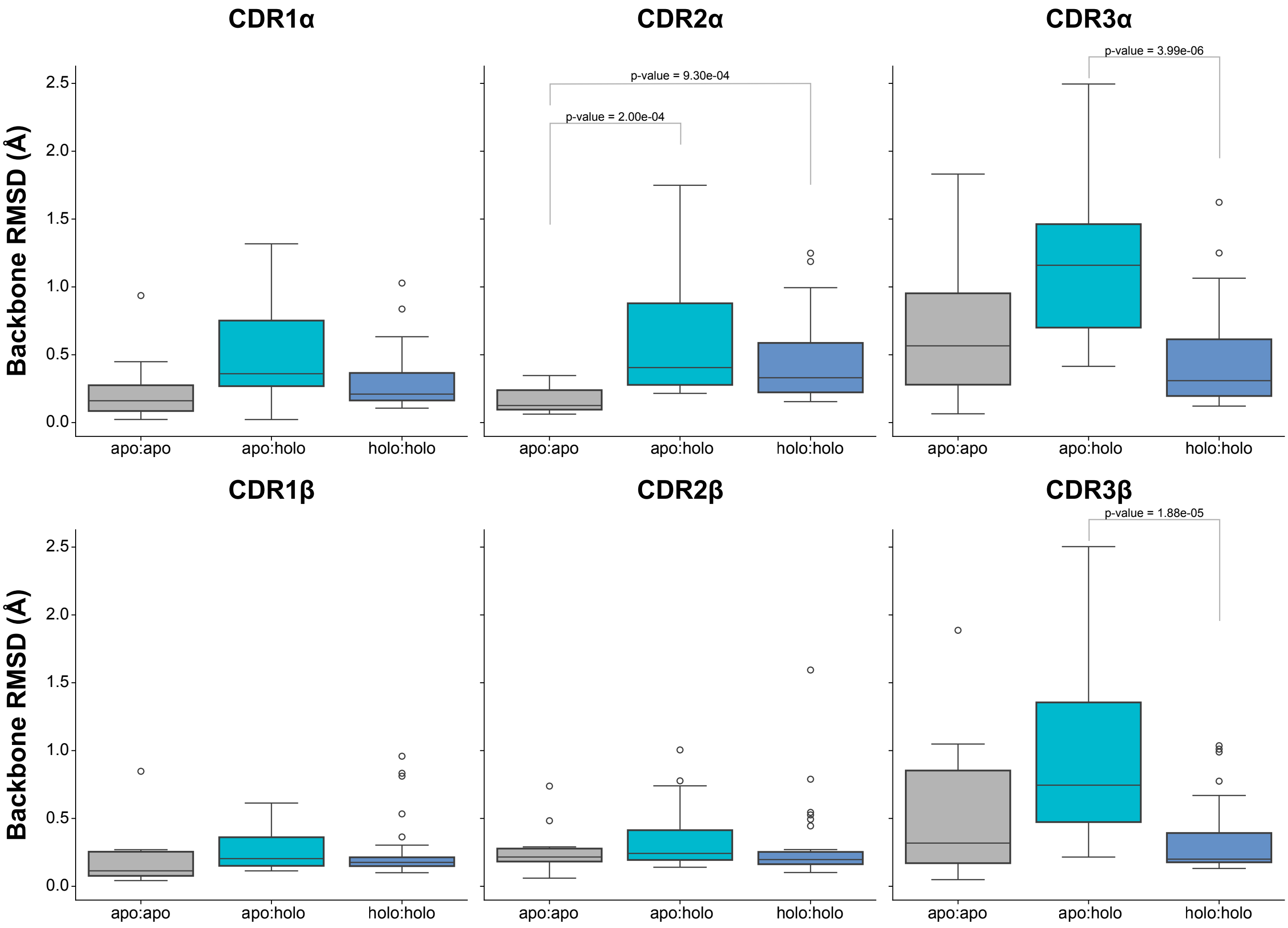

Figure S4. Comparison of different movement types for each CDR loop after alignment on loop backbones. *apo*:*apo* refers to changes between different *apo* structures of the same TCR, *apo*:*holo* refers to changes between *apo* and *holo* structures, and *holo*:*holo* refers to changes between different *holo* structures of the same TCR. There are significant differences between the movement types based on a p-value of 3.61×10^{-18} from a Kruskal-Wallis test (significance level < 0.05). Significant *post hoc* results have been added to the plot.

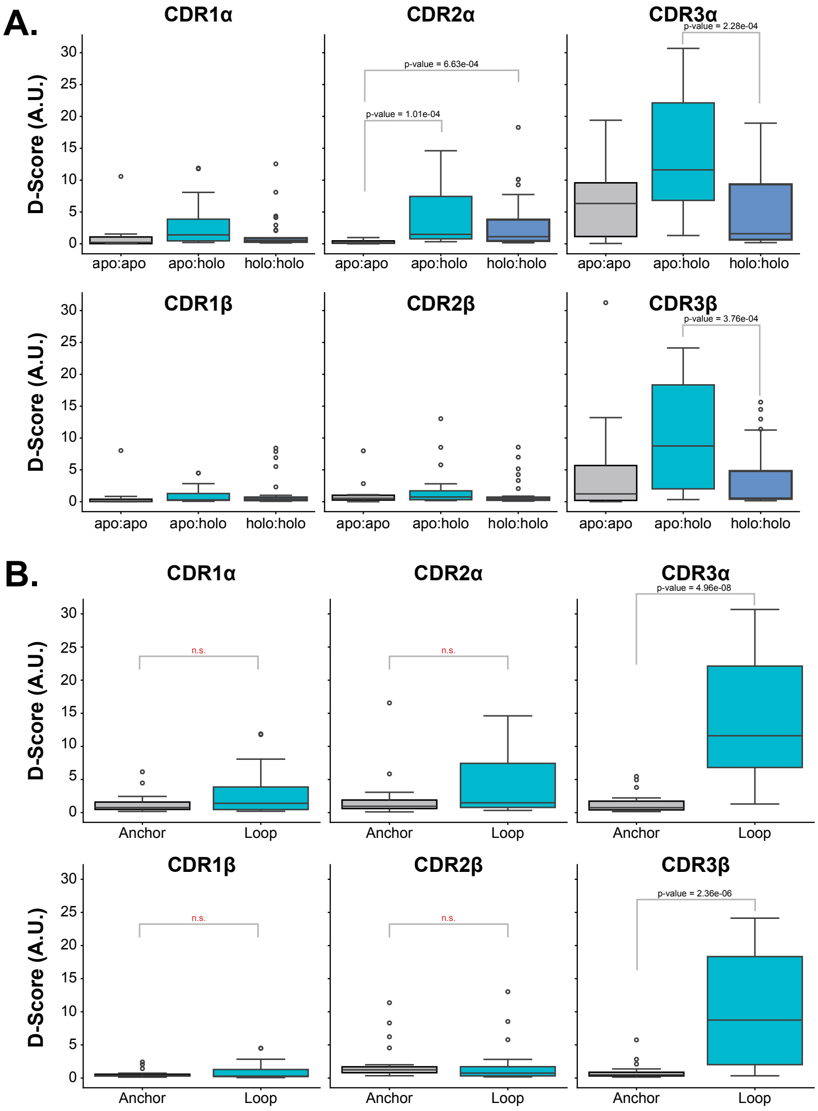

Figure S5. D-scores between different conformational states. **A.** Comparison of CDR D-scores between *apo*:*apo*, *apo*:*holo*, and *holo*:*holo* interactions. There are significant differences between the movement types based on a p-value of 3.57×10^{-18} from a Kruskal-Wallis test (significance level < 0.05). Significant *post hoc* results have been added to the plot. **B.** Comparison of CDR D-scores and anchor D-scores (for five anchor residues on either side of the CDR loop). There are significant differences between the regions based on a p-value of 1.51×10^{-16} from a Kruskal-Wallis test (significance level < 0.05).

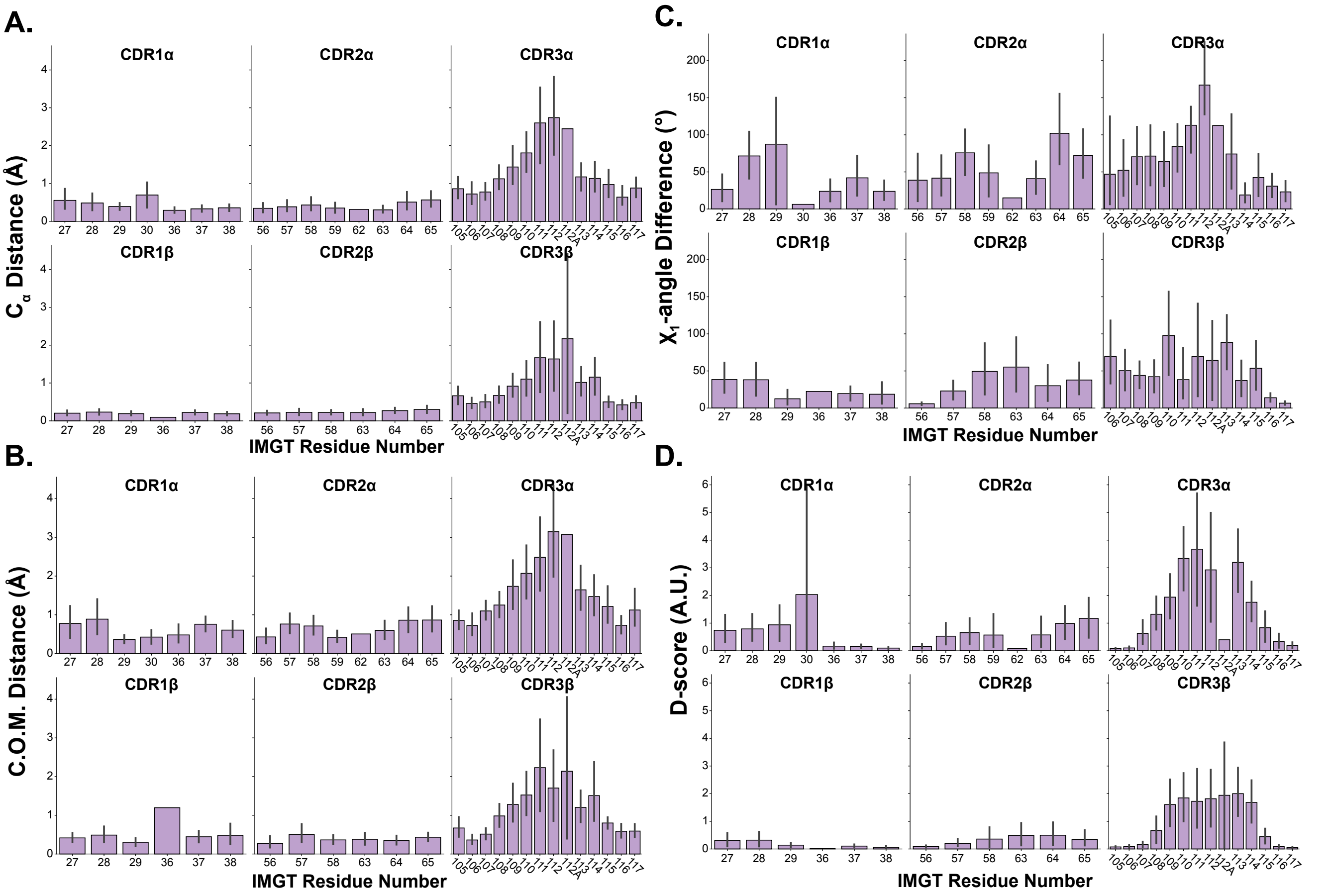

Figure S6. Describing the movement of each CDR loop in more detail after alignment on loop backbones. **A.** C_α movement between *apo* and *holo* conformations. **B.** Residue centre-of-mass changes between *apo* and *holo* conformations. **C.** χ_1-angle changes between *apo* and *holo* conformations. **D.** D-scores (backbone dihedral angle differences) between *apo* and *holo* conformations.

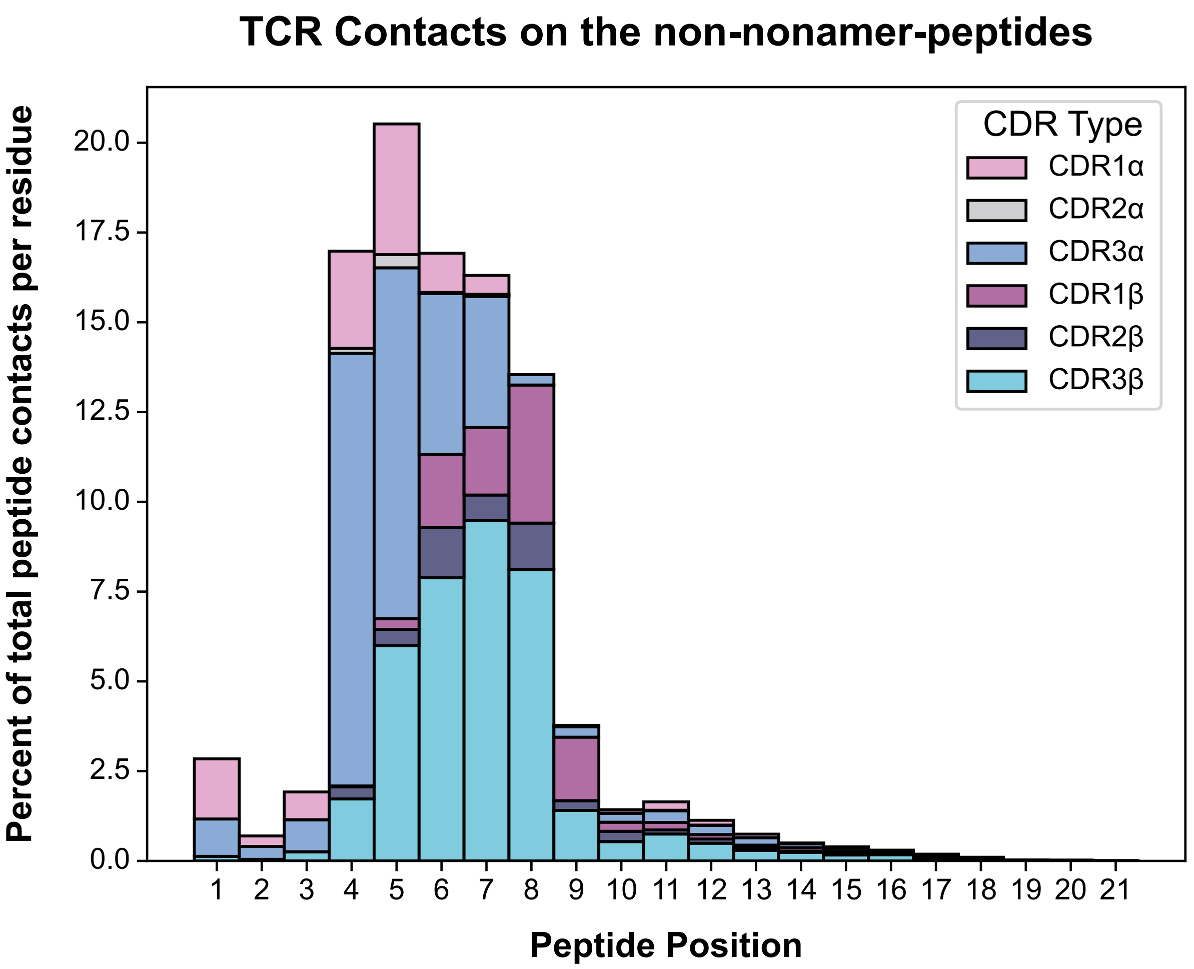

Figure S7. Distribution of contacts made between CDR loops and non-nonamer peptides from the STCRDab Leem et al. (2018) structures.

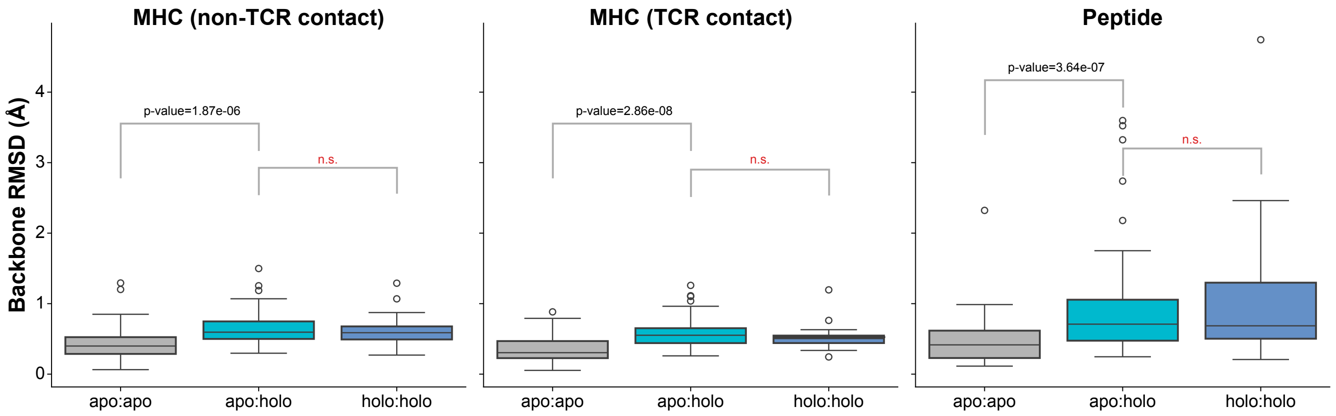

Figure S8. Comparison of *apo*:*apo*, *apo*:*holo*, and *holo*:*holo* changes for pMHC-Is. Significant differences exist between the different comparisons based on the results of a Kruskal-Wallis test at a 0.05 significance level (p-value 5.64×10^{-21}).

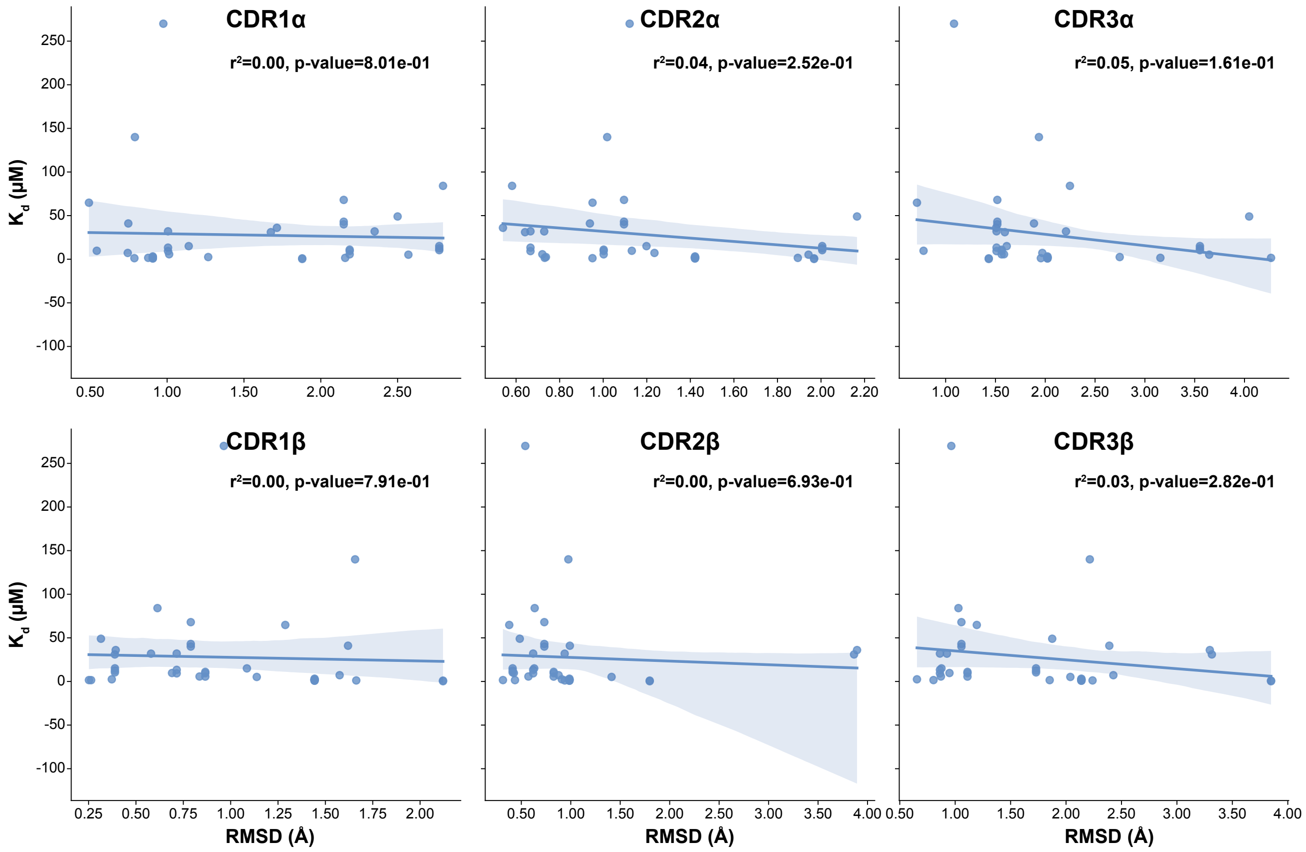

Figure S9. Correlating RMSD changes of CDR loops to affinity.

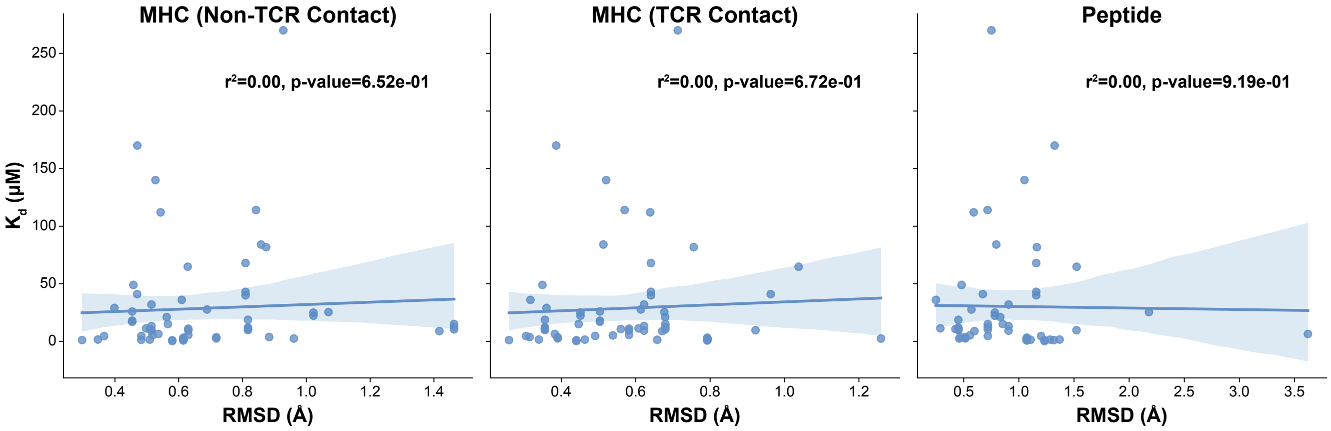

Figure S10. Correlating affinity changes of pMHC-Is to RMSD.

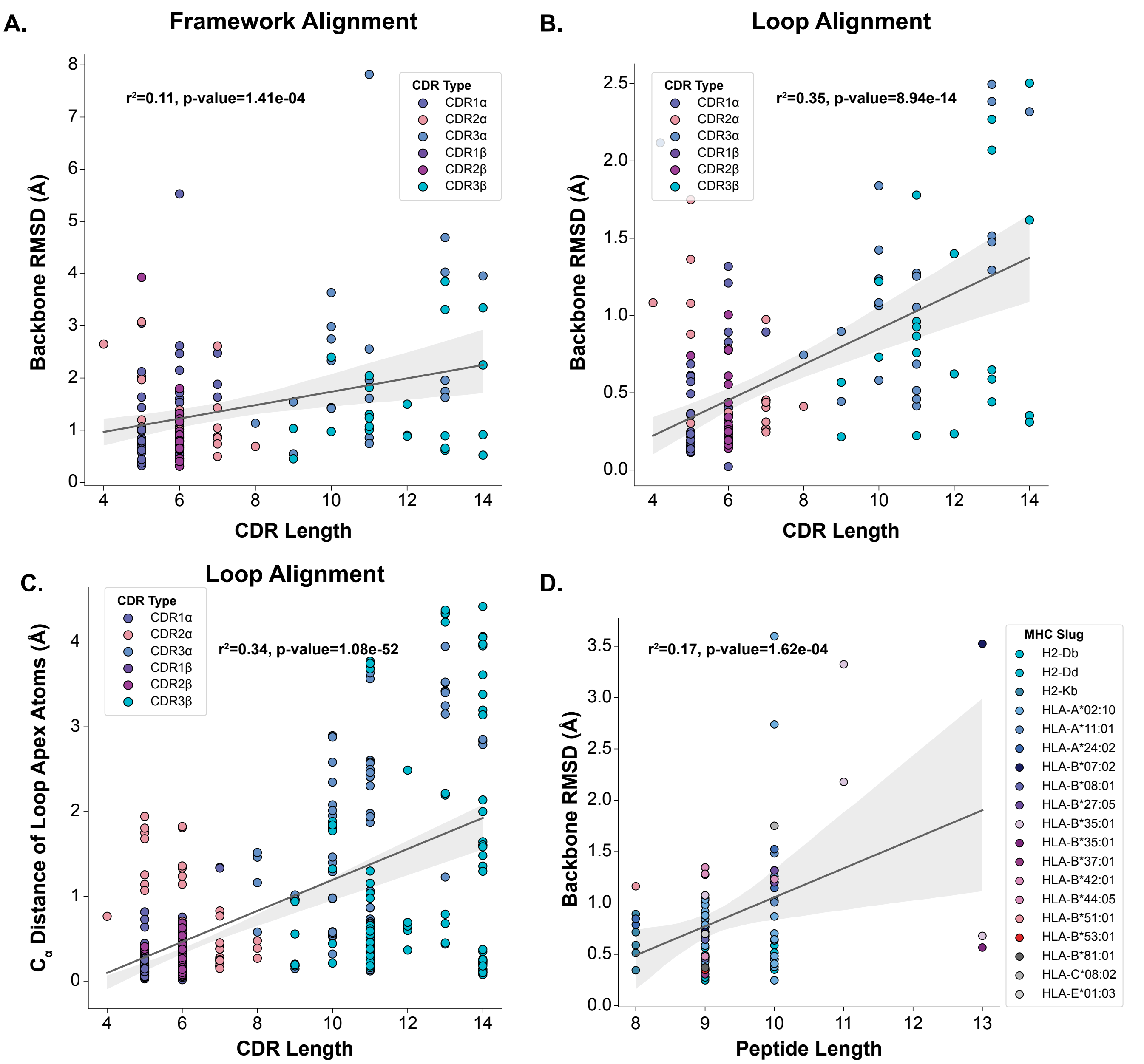

Figure S11. Correlation of CDR loop and peptide lengths (by number of amino acids) to conformational change. **A.** Correlation of CDR loops when RMSD conformational changes are measured after TCRs are aligned on framework regions. **B.** Correlation of CDR loops when RMSD conformational changes are measured after CDR loops are aligned before comparison. **C.** Correlation of the loop apex (C_α of middle residue for odd length loops or the average of the two middle C_α distances for even length loops) when CDR loops are aligned before comparison. **D.** Correlation of lengths and RMSD for peptides aligned on the floors of the MHC antigen binding domain.
